# Gender and Age-Related Decline in Lower Limb Standing Muscle Strength: Benchmarking for Rehabilitation Assessment

**DOI:** 10.3390/s26010069

**Published:** 2025-12-22

**Authors:** Vidas Raudonis, Viktorija Staneikaite, Ugnė Kubiliūtė, Raimondas Kubilius, Sarah Grube, Maximilian Neidhardt, Alexander Schlaefer, Gediminas Tankevičius

**Affiliations:** 1 Faculty of Electrical and Electronics Engineering, Kaunas University of Technology, LT-51367 Kaunas, Lithuania; 2 SustAInLivWork Center of Excellence, Kaunas University of Technology, LT-51367 Kaunas, Lithuania; 3 Department of Rehabilitation, Lithuanian University of Health Sciences, LT-44307 Kaunas, Lithuania; 4 Institute of Medical Technology and Intelligent Systems, Hamburg University of Technology, 21073 Hamburg, Germany; 5 Interdisciplinary Competence Center for Interface Research, 20246 Hamburg, Germany

**Keywords:** lower limb strength, sensor-based strength assessment, rehabilitation monitoring

## Abstract

This study aimed to demonstrate a novel sensor-based measuring stand for objective assessment of multi-directional lower limb muscle strength and to establish essential, age- and gender-stratified normative benchmarks. This cross-sectional study measured relative leg strength (N/kg) across six standing movements in 99 healthy, non-professional athletes (males and females aged 20–30, 40–50, and 60–70 years). Results confirmed that men exhibited significantly greater strength than women across all six directions (17% to 35% difference). Furthermore, a marked age-related decline was consistently observed in both sexes, with the largest and most clinically relevant differences (often exceeding 30%) concentrated in the transition to the 60–70-year range. Methodologically, these findings are limited to demonstrating age-related differences rather than longitudinal decline and are specific to an active, healthy cohort. This study demonstrates the sensor-based stand as an efficient, objective tool for comprehensive strength assessment, but its clinical utility is prospective and requires further validation against diverse and pathological patient populations.

## 1. Introduction

The assessment of lower extremity muscle strength is a fundamental component of physical medicine and rehabilitation, serving as a critical indicator of functional capacity, overall health, and quality of life. Leg muscle strength is vital for performing daily activities, maintaining balance, gait stability, and preventing falls, particularly as individuals age. A decline in muscle strength, known as sarcopenia, is closely linked to increased frailty, disability, and dependence [1,2]. Therefore, accurate and standardized methods for measuring muscle strength are essential for diagnosing conditions, monitoring rehabilitation progress, and evaluating the effectiveness of interventions.

Currently, leg muscle strength is often assessed through tests performed primarily in the sagittal plane (e.g., knee extension/flexion), which are important but may not fully capture the complexity of human movement [3]. Functional activities, such as walking, stair climbing, and changing direction, involve a complex interplay of forces across multiple planes, including the frontal plane (e.g., hip abduction/adduction). A comprehensive understanding requires evaluating strength across both the sagittal and frontal planes.

However, normative data for muscle strength often varies significantly across different age groups and genders, and standardized, accessible data that systematically stratifies these measures across both key planes (sagittal and frontal) for a broad population is frequently limited in clinical settings [4]. This lack of detailed, multi-planar normative data can complicate the accurate interpretation of muscle strength test results, potentially leading to misdiagnosis or suboptimal rehabilitation planning.

The current gold standard for lower limb strength assessment in rehabilitation often relies on manual muscle testing (MMT) or isolated isokinetic/handheld dynamometry. While valuable, these methods frequently lack the validity required to capture functional deficits during standing. significant gap exists in the literature regarding reliable, multi-directional standing strength norms—especially those stratified by age and gender and expressed as relative strength (N/kg). This deficiency limits the clinician’s ability to set objective, evidence-based recovery targets. Clinicians currently lack precise benchmarks to answer the relatively simple question: “How strong should this 65-year-old male or female patient be in hip adduction to ensure safe return to functional capacity?”. This study directly addresses this void by introducing a novel, multi-directional strength measurement system. By establishing detailed, stratified norms, we provide clinicians with the objective, performance-based reference values necessary to accurately quantify a patient’s deficit in strength. This article addresses this gap by aiming to systematically assess and document leg muscle strength in the frontal and sagittal planes across individuals of various ages and genders. By providing a detailed analysis of the differences and commonalities in muscle strength profiles within a diverse population, this work seeks to establish a more robust foundation for clinical evaluation. The findings will contribute valuable normative data, thereby enhancing the precision of diagnostic procedures and optimizing individualized treatment strategies in the fields of physical medicine and rehabilitation.

## 2. Recent Works

Muscle strength is universally acknowledged as a vital marker of health, with the literature establishing a clear link between lower extremity strength and crucial functional outcomes [5,6]. Strong leg muscles are essential for daily activities such as maintaining gait stability, rising from a chair, and climbing stairs. Deficits often correlate with reduced functional independence and increased reliance on assistance. Research confirms that age-related muscle loss (sarcopenia) is a major public health concern [7,8]. The literature highlights that muscle strength generally peaks in the third decade of life and declines progressively thereafter, making strength assessment a critical tool for identifying individuals at high risk for frailty and disability [9]. Numerous studies emphasize the predictive value of leg muscle strength—or lack thereof—in determining an individual’s risk of falling [10]. Targeted interventions based on strength assessments are paramount for fall prevention programs.

The measurement methodology utilizes various tools, but the literature shows a growing emphasis on precision and comprehensiveness. Isokinetic and isometric dynamometry remain the gold standards for reliable and objective strength measurement. These devices provide quantitative force data, minimizing subjective error. Traditional clinical practice often focuses heavily on strength in the sagittal plane (e.g., knee extension and flexion, which are vital for locomotion) [11,12]. However, the state of the art increasingly recognizes the critical role of the frontal plane (e.g., hip abduction and adduction) in lateral stability, dynamic balance, and preventing injuries [13,14]. The current literature suggests a lack of consistent, standardized data that concurrently evaluates strength in both planes, particularly outside of athletic populations. The literature draws a distinction between isolated strength measurements (using dynamometers) and functional tests (like the 30 s chair stand test or TUG test). The review emphasizes the need for isolated measurements to accurately quantify muscle deficits before linking them to functional performance [15].

Studies consistently report that males possess greater absolute muscle strength than females, primarily due to differences in muscle mass, hormonal factors, and body composition [16,17]. However, this analysis of the existing literature also notes that relative strength (strength normalized to body mass or muscle cross-sectional area) may show less significant gender disparities [18]. The literature confirms that strength decline related to age is not linear [19,20]. Strength changes must be meticulously assessed within specific age cohorts, as the rate and pattern of muscle degradation vary significantly. Establishing detailed age-stratified normative values for both the frontal and sagittal planes is important for accurate clinical comparison, which is a key gap identified in the existing body of knowledge.

In summary, the recent works confirm the necessity of objective leg strength assessment using dynamometry, which remains the gold standard for reliable quantification of force production. However, the existing clinical paradigm is critiqued for its over-reliance on the sagittal plane (movements like knee extension), which often neglects the critical role of the frontal plane (like hip abduction) in crucial daily functions such as lateral stability, maintaining balance, and dynamic gait control [21]. The literature therefore underscores the underrepresented importance of the frontal plane in comprehensive strength evaluation [22]. Furthermore, the variability introduced by demographic factors is substantial: strength norms differ markedly across age cohorts due to sarcopenia, and there are significant differences between genders [23]. Consequently, the consensus validates the need for comprehensive normative data that is stratified by both age and gender and includes measures from both the frontal and sagittal planes. Collecting and presenting this multi-layered data is the precise gap this article aims to fill, providing a more accurate basis for diagnosis and personalized rehabilitation planning.

## 3. Research Methodology

### 3.1. Subject Selection Criteria

**Figure 1:**
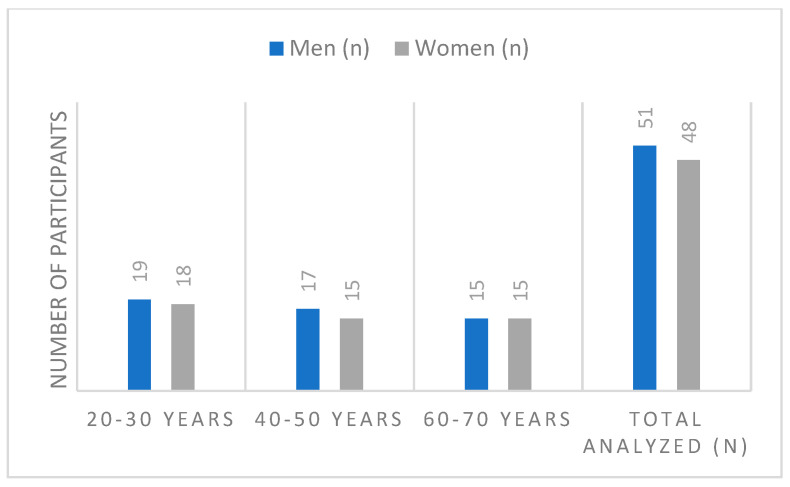
Distribution of participants according to age group.

The study focused on the relative leg muscle strength of non-professional athletes, both male and female, distributed across three distinct age groups: 20–30 years, 40–50 years, and 60–70 years. The core objective was to assess strength across different muscle chains activated by static movements performed in both the Frontal and Sagittal planes. Initially 108 participants and volunteers were involved for the study. Participants had to fall precisely into one of the three age groups (20–30, 40–50, 60–70 years) and must not be professionally involved in sports (see Figure 1). Nine people who participated but did not meet the exact age group criteria (i.e., ages 31–39 and 51–59) were excluded from the final data analysis (*n* = 9). The research was conducted over a period of five months, from November 2024 to March 2025.

The selection of the 20–30-, 40–50-, and 60–70-year groups was intentional and strategic. This method was adopted to create the maximal separation between cohorts, allowing us to clearly capture and model the age-related decline in strength across three distinct life stages—peak performance, mid-life, and early old age—for this initial normative study. The exclusion of participants falling in the intermediate decades (31–39 and 51–59) was a pragmatic choice to maximize the statistical contrast between the primary groups and ensure the most robust modeling. All participants were prescreened using exclusion criteria to ensure a healthy, non-clinical cohort. Only individuals with no known chronic or acute conditions affecting strength (musculoskeletal, neurological, etc.) were included. Any noted “health problems” were minor and clinically irrelevant to maximum isometric lower-limb strength.

### 3.2. Lower Limb Muscle Chain Strength

Traditional measures of muscular function, such as isolated maximal voluntary isometric contractions or isokinetic dynamometry, quantify the force output of a single muscle or muscle group. While fundamental, these tests often isolate the muscle in non-functional positions, failing to account for the crucial demands of real-world movement where force is generated through synchronized multi-joint action and superimposed postural control. We introducing and formally define the concept of lower limb muscle chain strength (MCS). MCS is a novel functional construct that measures the integrated, maximal force-generating capacity of a functional kinetic chain (trunk, hip, knee, and ankle) while simultaneously maintaining a specific posture. We present the methodological approach used to quantify MCS in six functional directions, providing the foundational evidence for its use in rehabilitation and functional assessment.

The measurement of lower limb MCS is inherently multi-joint and multi-planar. The six functional directions assess different combinations of muscle groups working isometrically to produce force while stabilizing the entire kinetic chain. Table 1 represents a breakdown of the primary muscles and joints involved in generating force for each of the six directions.

The primary challenge in evaluating lower limb muscle chain strength using a standard dynamometer (a gold standard) is that these devices are inherently designed to measure and isolate the force of a single joint, which is the inverse of the MCS construct. A standard isokinetic dynamometer would typically require the participant to be strapped into a chair to completely isolate a single joint. This setup completely removes the postural demand and multi-joint stabilization that defines MCS [24]. The device measures torque around a fixed axis, not the linear force (N) exerted by the entire chain while standing. Therefore, the two measurements assess fundamentally different constructs. Our claim is that this functional, stability-dependent capacity (MCS) is more clinically relevant to daily tasks than isolated strength.

### 3.3. Proposed Measurement Method and Procedures

The word “isometric” usually means holding a muscle perfectly still against an immovable object, like pushing on a wall while your body is held steady by a machine. Our novel “standing isometric test” is different. It is designed to capture a more complete, functional measure of how your muscles work in the real world. When a participant stands in a challenging, staggered position (Romberg-like) and pushes or pulls with maximum effort, the forces we record show more than just pure muscle strength. They show the combined effort of the muscle group and the participant’s own intrinsic balance and stability. Your core and leg muscles must instantly fire up to actively compensate and prevent you from losing balance due to your own force. This mirrors real-life demands—like trying to keep your balance while powerfully climbing a set of stairs. Since the test requires strength paired with dynamic stability, the data is highly relevant to functional performance and any related clinical deficits. The measuring system were custom-made primarily due to a specific functional requirement that existing commercial force platforms could not meet: the need to measure horizontal (push/pull) forces in both the anterior–posterior (sagittal) and medial-lateral (frontal) directions during single- and dual-leg standing isometric exercises.

**Figure 2:**
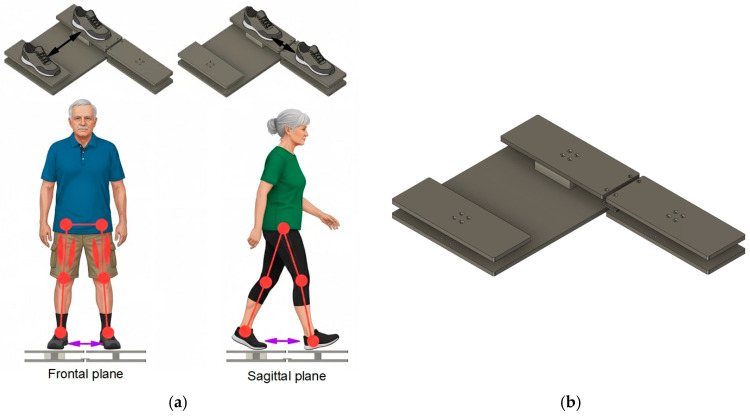
(**a**) Muscle strength measurements in the frontal and sagittal planes, (**b**) stance measurement platform with integrated 3D force sensor.

Muscle strength was assessed using a 3D force sensor which was integrated into a platform that the subject could stand on (see Figure 2b). The stance measuring platform is designed as a robust, low-profile base fabricated from aluminum alloy to ensure stability during static strength tests. Its primary component is the sophisticated system consisting of 3D force sensors and controllers integrated within the structure. These sensors rely on piezoelectric elements, which are crystals that produce an electrical charge proportional to the mechanical stress (force) applied. This technology allows the platform to accurately measure the ground reaction force in three dimensions (X, Y, Z) simultaneously. The platform is connected to a controller that transmits the high-precision force data to a laptop, where the results are preprocessed and visualized in real-time. This combination of a stable base and high-sensitivity piezo sensors allows for precise, objective measurement of the maximum force generated by the leg muscle chains.

The high-sensitivity triaxial (3D) force sensors measure participants stance by capturing the ground reaction force (GRF) in three orthogonal axes simultaneously, such as, vertical axis (*Fz*), mediolateral axis (*Fx*) and anteroposterior axis (*Fy*). *Fz* measures the vertical load, which is essential for determining a subject’s effective weight distribution and, in the context of the study, is used for normalizing strength into relative force (N/kg). *Fx* measures the force exerted from side-to-side. This is important for assessing balance and strength in the frontal plane, such as the adduction and abduction movements. *Fy* measures the force exerted from front-to-back. This is used for evaluating stability and static force in the sagittal plane (e.g., during Romberg-style forward/backward pushes).

**Figure 3:**
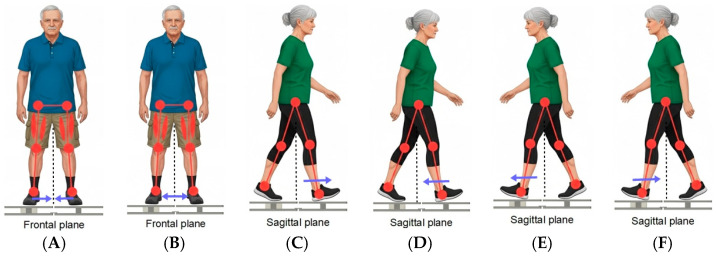
Visualization of exercises, where (**A**) represents direction A, (**B**) direction B, (**C**) direction D1, (**D**) direction D2, (**E**) direction K1 and (**F**) represents direction K2.

All measurements were performed while the subject was standing on the platform, executing specific static movements to generate the maximum force output from various leg muscle chains (see Figure 2a). The forces were measured during static pushes where the participant stood on the platform and attempted to push or pull against the force plate structure. This methodology isolates the muscle chain’s maximum strength capacity in specific movement vectors, such as, side-to-side static movements in frontal plane and forward-backward movements in sagittal plane. Side-to-side directions primarily target the abductor and adductor muscle chains of the leg, which are essential for lateral stability and gait mechanics. Participants were asked to perform fallowing static motions standing on the platform:

1. Direction A (Adduction). Force was measured while the subjects attempted to pull their legs inward from the sides toward the center. This assesses the maximum strength of the inner leg muscle chains (see Figure 3A);
2. Direction B (Abduction). Force was measured while the subjects attempted to push their legs outward from the center to the sides. This assesses the maximum strength of the outer leg muscle chains (abductors), particularly the gluteus medius (see Figure 3B);
3. Direction D1 (Right-Forward Push). Force was measured when the subject pushed forward from the body’s center, with the right leg placed in front (see Figure 3C);
4. Direction D2 (Right-Backward Pull). Force was measured when the subject pulled backward toward the body’s center, with the right leg placed in front (see Figure 3D);
5. Direction K1 (Left-Forward Push). Force was measured when the subject pushed forward from the body’s center, with the left leg placed in front (see Figure 3E);
6. Direction K2 (Left-Backward Pull). Force was measured when the subject pulled backward toward the body’s center, with the left leg placed in front (see Figure 3F).

The study assessed lower limb muscle strength using six standardized static standing exercises, designed to evaluate functional muscle chains in both the frontal and sagittal planes. The measurements are taken in six standardized static standing exercises to assess lower limb muscle chain strength in both the frontal and sagittal planes. These tests isolate the functional capacity of different muscle groups while the participant maintains a stationary, standing posture against the measuring stand. These two directions measure the lateral strength of the muscle chains responsible for stabilizing the body side-to-side, primarily involving the hip abductors and adductors (see Table 2).

Sagittal plane strength assessment in done in four directions. These four directions assess the anteroposterior (forward/backward) strength of the lower limb muscle chains, which are critical for controlling movement in the standing and walking phases. To isolate these forces, the measurements require the participant to adopt a Romberg-like, staggered stance where one foot is placed in front of the other on the force-measuring platform. The four directions are based on which leg is forward and the direction of the applied force. In direction D1 participant stands with their right leg (D) placed forward. They generate maximum isometric force is measured by pushing away from the body center to the front (anteriorly). This primarily assesses the Anterior muscle chains (extensors like the quadriceps and plantar flexors) of the front leg, along with the stabilizing role of the rear leg’s muscle chain. Maintaining the right leg (D2 direction) forward, the participant generates maximum isometric force by pulling the sensor from the front toward the body center (posteriorly). This assesses the Posterior muscle chains (flexors like the hamstrings and gluteals) of the front leg and the stabilizing function of the rear leg. The participant stands with their left leg (K) placed forward (direction K1). They generate maximum isometric force by pushing away from the body center to the front (anteriorly). This assesses the anterior muscle chains (extensors) of the front leg and the stabilizing chain of the rear leg. Maintaining the left leg (direction K2) forward, the participant generates maximum isometric force by pulling the force from the front toward the body center (posteriorly). This assesses the Posterior muscle chains (flexors) of the front leg and the stabilizing chain of the rear leg.

**Figure 4:**
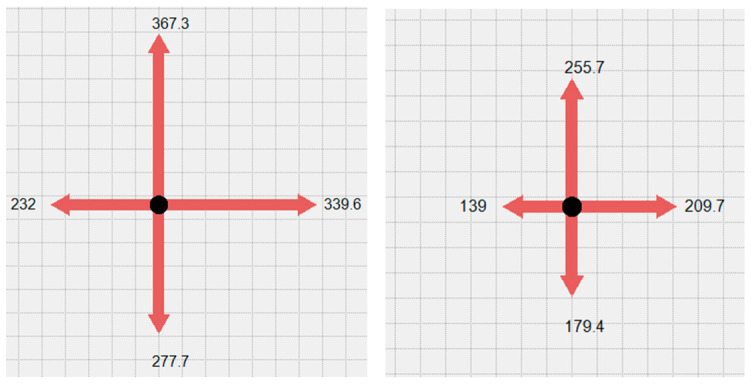
Example of visualization of generated force shown in Newtons.

To ensure participants reached true maximal voluntary isometric contraction and to enhance data reliability, a standardized protocol was implemented. For each of the six testing directions (A, B, D1, D2, K1, K2), participants performed three maximal isometric efforts. Each effort required the participant to ramp up to and sustain their maximum push or pull for a minimum of 1.5 s (more in Section 3.4), accompanied by consistent verbal encouragement from the researcher. Following a one-minute rest period between attempts for the same direction, the data was analyzed. In line with established isometric strength testing principles, the highest force value recorded across the three trials was selected and used as the definitive maximal strength output for that specific muscle chain assessment. The platform’s sensors registered the maximum force produced in Newtons (N) for each of these six distinct, static isometric movements. Verbal encouragement was given to motivate the subjects. The researcher registered the results in real-time on a laptop screen, which was visible to both the researcher and the participant (see Figure 4).

### 3.4. Signal Acuisition

The sensor unit in the platform records a continuous analog electrical signal that is proportional to the mechanical force applied to it. Specifically, the piezoelectric elements generate a voltage or charge signal for each of the three dimensions (*Fx*, *Fy*, *Fz*). This raw electrical output is then converted by an analog-to-digital converter into a digital signal that is sent to the computer. The ultimate recorded data stream consists of time-series force values (in Newtons) for the vertical, medial/lateral, and anterior/posterior directions.

The core component, the triaxial force sensor, utilizes three independent Wheatstone bridges (one for each *Fx*, *Fy*, *Fz* axis). The applied force causes proportional changes in the resistance of the internal strain gauges. The bridge, powered by a stable excitation voltage (e.g., 10 V), outputs a corresponding, very small differential analog voltage signal, with the maximum useful signal being approximately 10 mV. Since this raw signal is too weak, it is fed into specialized signal conditioning circuits (Instrumentation Amplifiers). The amplifiers provide the necessary gain (approximately 360 times), to amplify the 10 mV signal up to the 3.6 V input range of the microcontroller’s analog-to-digital converter (ADC).

The input power supply limits are set between 12–35 V. The force sensor’s sensing element is powered by a 10 V voltage. The necessary voltage reduction is achieved using the adjustable low-dropout positive voltage regulator LD1117 from STMicroelectronics. This voltage is expected to be maintained within tight fluctuation limits. Typically, the regulator’s output voltage is adjusted using two external resistors, *R*_1_ and *R*_2_, based on a standard Formula (1):

(1) VOUT=VREF(1+R2R1)

**Figure 5:**
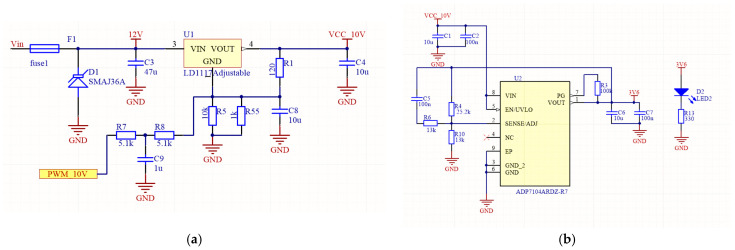
(**a**) Electrical diagram of voltage regulator with closed-loop control for 10 V supply and (**b**) electrical diagram for 3.6 V power supply.

Real-time voltage regulation was required in stance measurement platform, which was implemented by introducing a closed-loop feedback circuit. This loop consists of a 10 V measurement subsystem and the 10 V output voltage regulator controlled by a pulse-width modulation (PWM) circuit (see Figure 5a). By selecting appropriately rated circuit resistances, the LD1117 regulator’s output voltage is dynamically regulated within the narrow range of [9.950 V; 10.050 V], dependent on the PWM duty cycle. The PWM period is chosen as 13.8 μs and uses 1000 counts (steps). In this way, the resolution for the 10 V regulation is 100 μV per step.

The input voltage of the selected microcontroller (MCU) is in the range of [2.8–3.6 V]. This same voltage is utilized to power the ADC. To maximize the ADC’s measuring range, a 3.6 V supply voltage was selected. The required 3.6 V is generated using an LDO-type DC/DC step-down converter, the ADP7104ARDZ (see Figure 5b). This specific component was chosen due to its low noise characteristics, sufficient power capacity, and the capability to precisely regulate the output voltage, which is crucial for maintaining the stability and accuracy of the ADC reference. The differential signal from one axis of the 3D force sensor is amplified by the INA188 amplifier. The output signal of this amplifier *V_OUT_* is calculated by Formula (2).

(2) $$V_{OUT}=G\;\left(V_{+}-V_{-}\right)+V_{REF}$$

where *V_OUT_*—is the output signal, *G*—is the grain coefficient, $V_{+}$—is the positive input signal, $V_{-}$—is the inverted input signal and *V_REF_*—is the reference voltage signal (offset).

Since the sensor’s axis is designed to measure force in both tension (positive) and compression (negative) directions, the amplifier’s output signal must vary between a negative rail (−*V_DD_*) and a positive rail (+*V_CC_*). Because the circuit uses a single-polarity supply, a fixed offset *V_REF_* was introduced to shift the entire signal range so that the zero-force condition corresponds to a positive voltage, typically half the supply voltage 1.8 V. The *V_REF_* signal is formed from the microcontroller’s PWM signal, integrated using an RC filter network, and then buffered by an operational amplifier to reduce output impedance.

Calibration is performed, because manufacturing imperfections cause the sensor’s zero-force output to deviate slightly from true zero. The offset is individually set for each sensor’s axis by reading measured values while the sensor is unloaded. By adjusting the PWM duty cycle, the *V_REF_* value is controlled until the instrumentation amplifier’s output registers precisely half of the supply voltage. The reference signal value is controllable within the range of 0 V to 2.32 V. Using a 72 MHz clock frequency PWM signal with a period of 50,000 counts, the *V_REF_* signal is controlled with a high resolution of 0.005 mV.

The STM32F373CB microcontroller was selected for controlling whole stance measuring system. This is a 32-bit RISC architecture processor equipped with an FPU, an MPU, and 128 kB of Flash memory. The most critical reason for choosing this specific microcontroller is its integrated 16-bit ADC, in addition to its compact package and required pin count. STM32 microcontrollers typically employ 12-bit SAR (Successive Approximation Register) type ADCs, where the Least Significant Bit (LSB) at a standard 3.3 V supply is 0.8 mV. Conversely, the 16-bit converter offers a theoretical LSB of only 0.05 mV. The declared noise-free resolution for this unit is 14 bits, which corresponds to an LSB of 0.2 mV. Consequently, utilizing the ADC achieves at least 4 times better resolution than the standard SAR type. The data transmission to the computer is managed using the RS-485 interface. The procedural flow of the system program is shown below (Algorithm 1).
**Algorithm 1.** The procedural flow of the system program.**WHILE**:   1. *CHECK STATUS*: *IS DATA TRANSMISSION STOPPED*?   **IF NOT** data_transmission_active:    data_transmission_active = True **   ELSE**   2. *DATA STREAMING MODE*: *Continuous Measurement*    2.1 Acquire Raw Data      raw_readings = read_sensor_data_adc()    2.2 Process Data      avg_adc_value = calculate_average(raw_readings)    2.3 Apply Calculations force to Newtons      force_data_newtons = calculate(avg_adc_value, calibration_coefficients)    2.4 Transmit Data      send_data_rs485(force_data_newtons)    2.5 Check for STOP command during streaming   **ENDIF** **ENDWHILE**

In the main program loop, all utilized ADC channels are read, supply voltages are regulated, compression/tension forces are calculated, and data exchange with the computer organized. The main program loop calculates the measured voltages in millivolts and converts them into Newtons (*F*) for all axes using Formula (3).

(3) $$F(N)=\left({ADC}_{Value}-{Zero}_{Offset}\right)\times {Gain}_{Factor}$$

where ${Gain}_{Factor}$ is a fixed value calculated during the calibration process when the force sensors are loaded with a known reference weight. ${Zero}_{Offset}$ in our case is equal to 1800.

### 3.5. Signal Preprocessing

The maximum force produced during each static isometric movement (push or pull) is not determined by a single peak reading, but rather through a process of continuous data smoothing and window analysis. This method is important for ensuring the recorded values (*F_x_*, *F_y_* and *F_z_*) accurately represents the subject’s sustained maximum voluntary contraction (MVC) and not a transient measurement error. The device records data at a frequency of 1.4 kHz, generating 1400 data points every second. The raw force data signals were subjected to filtering to remove high-frequency noise and artifacts resulting from physiological tremor and system vibration. The data was processed using a Butterworth low-pass filter with a cut-off frequency of 15 Hz. This setting ensures that the relevant muscle force activity (which typically operates well below 10 Hz) is retained, while unwanted signals are effectively removed, thereby enhancing the reliability of the maximal force detection.

**Figure 6:**
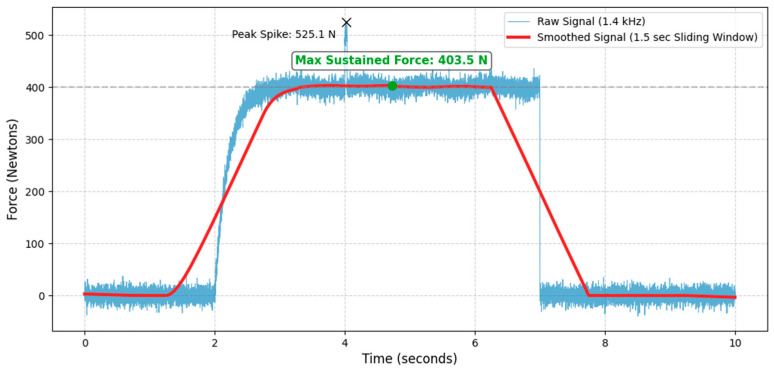
An example of sustained maximum voluntary contraction measured at 403.5 N.

When a subject performs a push, the recorded force signal is inherently noisy due to muscle tremor, minor balance shifts, and potential electrical artifacts. To mitigate these issues, additionally a sliding window average of 1.5 s is employed. A sliding window average smooths out high-frequency spikes and fluctuations. This averaging process ensures that quick, momentary spikes—which do not reflect true muscular capacity but rather sudden impacts or artifacts—are suppressed. The window size of 1.5 s requires the subject to maintain their maximum effort for a sufficient duration. The system does not just look for the single highest point in the entire trial; instead, it identifies the highest mean force value recorded within any continuous 1.5 s segment of the trial. Since the subjects are given verbal encouragement to motivate them to push as hard as possible, the trial typically extends beyond 1.5 s. The sliding window continuously moves across the force trace, ensuring that even if the absolute peak force occurred, for instance, between the 2.0 s and 3.5 s marks, that sustained segment is captured and validated as the true maximum (example is in the Figure 6).

## 4. Experimental Implementation and Results

This research successfully employed a platform-mounted digital 3D force sensor to investigate the leg muscle strength of 99 analyzed participants (from a total of 108) of various ages and genders. Strength was measured in six directions across the frontal and sagittal planes (as explained in Section 3.2). The data was processed and presented as relative force (N/kg), providing a robust, weight-normalized metric suitable for benchmarking.

Data were processed using the IBM SPSS Statistics 29 software program. Descriptive statistics were applied for analysis: means *M* and standard deviations SD were calculated. To compare the results between men and women, an independent samples *t*-test was used, with the differences in means expressed as percentages. The percentage difference between means was calculated using Formula (4).

(4) $$Difference\;(\%)=\frac{M_{Group1}-M_{Group2}}{M_{Group2}}\times 100$$

where *M* represents the means of the groups being compared. A *p*-value of <0.05 was considered as statistically significant.

### 4.1. General Evaluation of Participants

In the 20–30-year age group, there were more men (51.4%) than women (48.6%). Similarly, in the 40–50-year age group, there were also more men (53.1%) than women (48.9%). In the 60–70-year age group, the subjects were distributed equally by sex. Comparing the age groups, the 20–30-year group constituted 37.4% of the entire sample, the 40–50-year group made up 32.2%, and the 60–70-year group represented 30.3% of the total population.

The average height for women across all age groups was similar, at approximately 168.65 cm SD = 0.30. Men’s height varied: the G25 group (men 20–30 age group) had the highest average height at 186.47 cm. The G45 group’s (men 40–50 age group) average was only 0.12 cm less than the G25 group. The V65 group’s (men 60–70 age group) average height was 177.27 cm, a difference of 9.2 cm from the tallest group (G25).

Regarding average age, the male subjects in the G25 group were, on average, 23.26 years old (SD = 2.446), while the women in the same age group (G25) were, on average, 24.44 years old (SD = 3.072). The youngest subject was 21 years of age. In the G45 group, male subjects were, on average, 44.76 years old (SD = 3.580), and the average age for the M45 group was 46.13 years (SD = 3.852). The G65 group’s average age was 65.87 years (SD = 2.924). The average age of participants in the M65 group was 62.87 years (SD = 2.825), with the oldest subject in this group being 68 years old. The anthropometric characteristics by group are presented in Table 3.

When subjects were asked whether they had any health problems that could have influenced the research results, the highest percentage of health issues was recorded in the G25 group (women 20–30 years) at 22.22% (*n* = 4), and the G45 (*n* = 3) and G65 (men 60–70 years) groups, both at 20% (*n* = 3). The G45 and G25 groups (men) reported the fewest health issues, both at only 5.26% (*n* = 1) each.

### 4.2. Generated Force Differences Between Men and Women, Regardless of Age

To evaluate the generated force differences between men and women regardless of age, an independent samples *t*-Test was used. The *t*-test was performed based on the average relative force indices N/kg collected from all age groups. The study sample consisted of men (*n* = 51) and women (*n* = 48). The variable analyzed was the force measurement results performed by the subjects. Analyzing the statistical data revealed that the results for all six movements were statistically significant (*p* < 0.05), and the force produced by men was greater than that of women. All mean results for men were higher than for women, ranging from 17.00% (Direction B) to 31.70% (Direction A), depending on the measurement direction.

**Figure 7:**
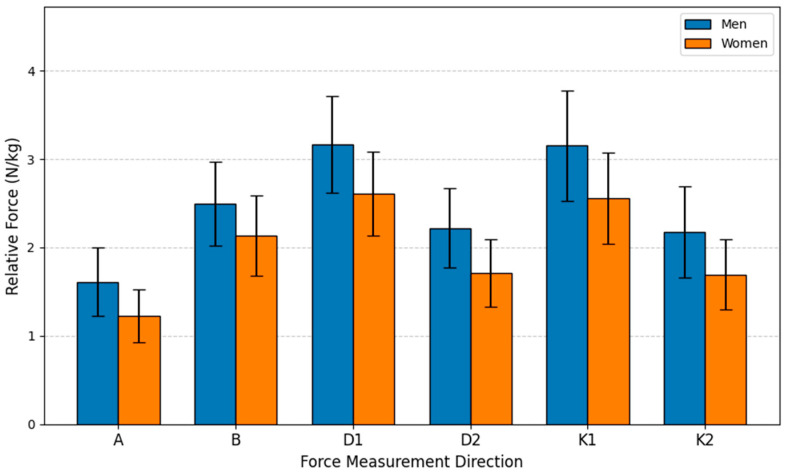
Relative Force (N/kg) by sex and direction.

The bar graph visually presents the average relative isometric lower extremity force (N/kg) for men and women across six distinct measurement directions (A, B, D1, D2, K1, K2) (see Figure 7). The primary conclusion is that men demonstrated statistically significantly greater force than women in all six movements, irrespective of age group, as indicated by the highly significant statistical markers (*p* < 0.001). This disparity highlights a fundamental sex-based difference in normalized strength capacity. Both groups recorded their highest overall mean forces in the D1 and K1 directions. The consistent pattern across all lower body isometric tests confirms the strong influence of biological sex on relative strength metrics, despite normalizing the force to body mass.

### 4.3. Generated Force Differences Between Men and Women in the 20–30 Year Age Group

This study aimed to evaluate the force differences between men and women in the 20–30-year age group. The sample included men (*n* = 19) and women (*n* = 18). In analyzing the differences between men and women in the G25 group, an independent samples *t*-Test was used, following the same principle as in previous section. Analyzing the statistical data revealed that the results were statistically significant for all motion directions. All mean results for men were higher than for women, ranging from 16.23% (Direction B) to 35.47% (Direction A), accordingly.

**Figure 8:**
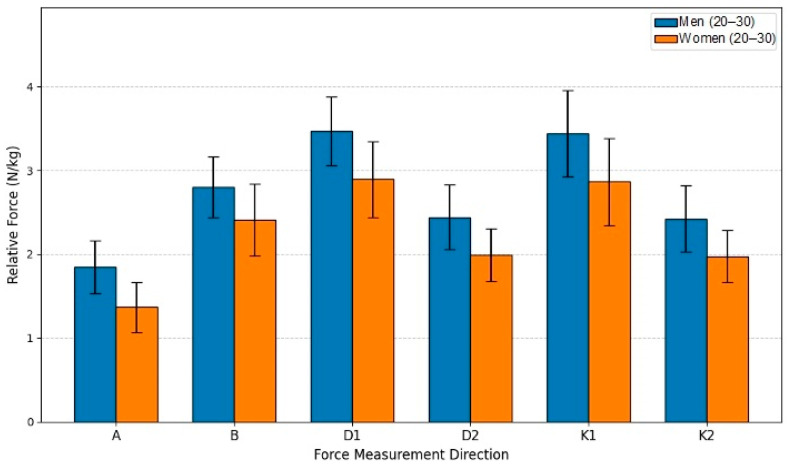
Relative Force (N/kg) by sex and direction in age group G25.

The bar graph (see Figure 8), based on the statistical analysis of the G25 age group, clearly demonstrates a statistically significant advantage in relative lower extremity isometric force (N/kg) for men across all six measurement directions. The independent samples *t*-test confirmed that every comparison between the male and female groups yielded a statistically significant difference, with the majority of movements reaching the highest level of significance (*p* < 0.001). Notably, both groups achieved their highest relative force values in Directions D1 and K1, suggesting these specific movement patterns allow for the greatest muscle recruitment efficiency relative to body mass. Overall, the data underscores a consistent, inherent biological difference in strength metrics within the young adult population, even after normalizing force to body mass. The largest force disparity was observed in Direction A (35.47% advantage; *p* < 0.001), where the mean force for men (1.8504 N/kg) significantly exceeded that of women (1.3659 N/kg). The smallest difference was recorded in Direction B (16.23% advantage; *p* = 0.005). All remaining measurements (D1, D2, K1, K2) showed men’s force to be between 19.79% and 22.80% greater than women’s, with all *t*-tests indicating statistically significant differences. This robust finding highlights the pervasive influence of sex on relative strength capabilities, even among young adults.

### 4.4. Generated Force Differences Between Men and Women in the 40–50 Year Age Group

**Figure 9:**
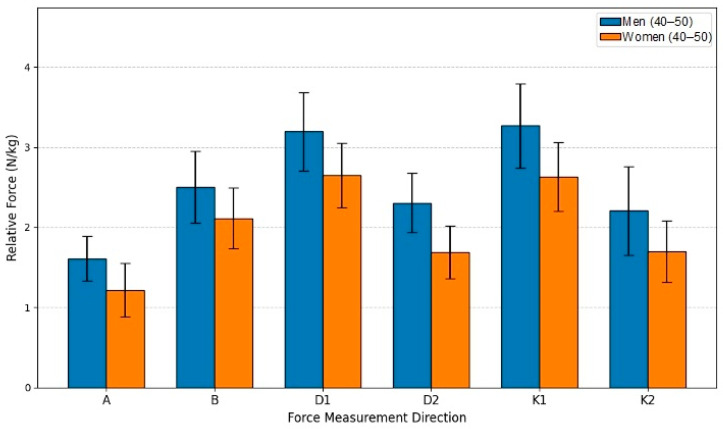
Relative Force (N/kg) by sex and direction in age group G45.

This study aimed to evaluate the relative isometric lower extremity force differences between men (*n* = 17) and women (*n* = 17) within the 40–50-year age group. Utilizing an independent samples *t*-test for comparison, the analysis established that men produced statistically significantly greater force than women in all six measured directions (*p* < 0.05). The force advantage held by men consistently ranged from 18.37% to 36.25% across the movement directions. The largest difference was found in Direction D2, where the men’s mean force (2.3039 N/kg) was 36.25% higher than the women’s mean (1.6909 N/kg), achieving high statistical significance (t(30) = 4.893, *p* < 0.001). A substantial difference was also observed in Direction A, with men showing a 32.40% advantage (*p* < 0.001). The smallest, though still significant, difference was in Direction B (18.37% advantage *p* = 0.014). Collectively, these results confirm that the sex-based disparity in relative strength remains a robust finding even in the middle-aged cohort. The mean force values for men in all six directions exceeded those of women, reinforcing the conclusion that biological sex is a dominant factor influencing relative isometric strength (see Figure 9).

### 4.5. Generated Force Differences Between Men and Women in the 60–70 Year Age Group

This study aimed to evaluate the relative isometric lower extremity force differences between men (*n* = 15) and women (*n* = 15) within the 60–70-year age group. The analysis employed an independent samples *t*-test to compare the mean relative force (N/kg) between the sexes across six different movements. The statistical analysis revealed that the differences were statistically significant for all six measurements, confirming a pervasive influence of sex on strength even in this older adult cohort. In every movement direction, the men’s mean force was consistently higher than the women’s, with the male advantage ranging from 15.41% to 34.30%.

**Figure 10:**
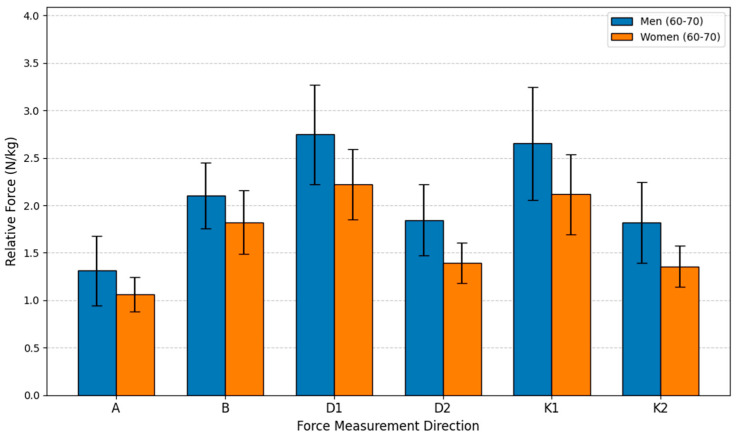
Relative Force (N/kg) by sex and direction in age group G65.

The most substantial advantage for men was recorded in Direction K2, where their force was 34.30% greater than the women’s, achieving high statistical significance (*p* < 0.001). A similarly large difference was found in Direction D2, with a 32.38% advantage for men (*p* < 0.001). The smallest disparity was observed in Direction B, where men’s force was 15.41% greater (*p* = 0.031). Directions A, D1, and K1 also showed significant differences, with men exhibiting force advantages of 23.43%, 23.68%, and 25.27%, respectively (*p* ≤ 0.026 for all). These results indicate that while relative force generally declines with age, the fundamental sex-based difference in strength capacity is maintained, with older men retaining a statistically significant and substantial advantage over older women in these specific lower body isometric movements (see Figure 10).

### 4.6. Generated Force Differences Among Women When Comparing Across Various Age Groups

The evaluation of the changes in relative lower extremity isometric force (N/kg) across different female age cohorts, a one-way analysis of variance (ANOVA) followed by the Games-Howell post hoc test was performed. The analysis compared three groups: 20–30 years old, 40–50 years old, and 60–70 years old across six distinct force measurement directions (A, B, D1, D2, K1, K2). The overall ANOVA results confirmed statistically significant differences between the three age groups in all six directions (*p* < 0.05 for all), indicating that age significantly impacts relative force capacity.

The subsequent Games-Howell post hoc analysis clarified the pairwise comparisons, revealing a clear pattern of age-related decline. The most consistent and pronounced differences were found when comparing the oldest group G65 to the two younger groups. For Direction A, D1, and K1, statistically significant differences were established between the G25 and G65 groups, and between the G45 and G65 groups, but the difference between the G25 and G45 groups was non-significant (*p* > 0.05), suggesting relative force is well-maintained up to middle age in these specific movements before dropping significantly in the 60–70-year bracket. However, two directions, D2 and K2, demonstrated a more gradual, but consistent, decline. In these two movements, the Games-Howell test showed statistically significant differences across all three paired comparisons G25 vs. G45, G25 vs. G65, and G45M vs. G65M, indicating a steady loss of relative force starting earlier than the 60 s.

**Figure 11:**
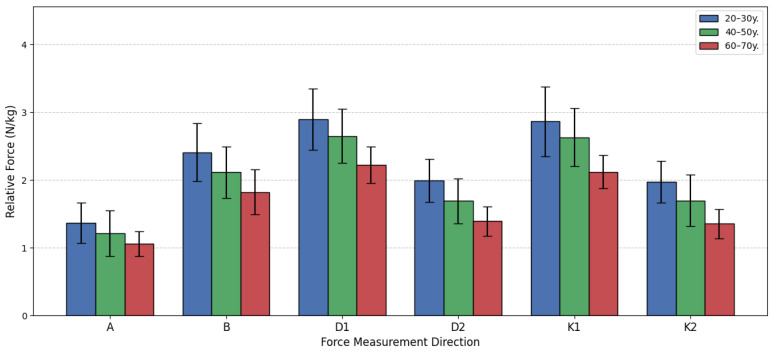
Women’s relative force differences by age group.

Quantifying the decline, the youngest group exhibited the highest force across the board. Their advantage over the G65 group was substantial, reaching a peak of 45.61% greater force in Direction K2 and 42.79% greater force in Direction D2. Even the G45 group maintained a strong advantage over the G65 group, with force metrics ranging from 14.37% to 25.21% higher. These findings strongly support the conclusion that relative lower extremity force is progressively diminished with age, with the most dramatic decline occurring after the 50 s. Figure 11 demonstrates the bar graph of women’s relative force differences by the age group.

### 4.7. Generated Force Differences Among Men When Comparing Across Various Age Groups

This section presents the comparison of relative lower extremity isometric force among three male age groups (see Figure 11). The data was analyzed using a one-way analysis of variance (ANOVA) followed by the Games-Howell post hoc test, applying the same statistical principles as used for the female groups. The results consistently indicate that as age increases, the men’s relative force declines across all measured directions. The most significant differences were typically observed when comparing the oldest group G65 to the two younger cohorts G25 and G45, while the differences between the young G25V and middle-aged G45V groups often did not reach statistical significance (*p* > 0.05). This suggests that a substantial decline in relative strength capacity predominantly occurs after the age of 50. For instance, in Direction A, the ANOVA showed a significant overall difference (F(2, 48) = 11.840, *p* < 0.001). Post hoc testing confirmed significant differences between G25 and G65 (*p* < 0.001), and between G45V and G65 (*p* = 0.043). The G25V group was 40.97% stronger than the G65 group, and the G45V group maintained a 22.63% advantage over the G65 group. However, the difference between G25V and G45V narrowly missed the significance threshold (*p* = 0.052).

**Figure 12:**
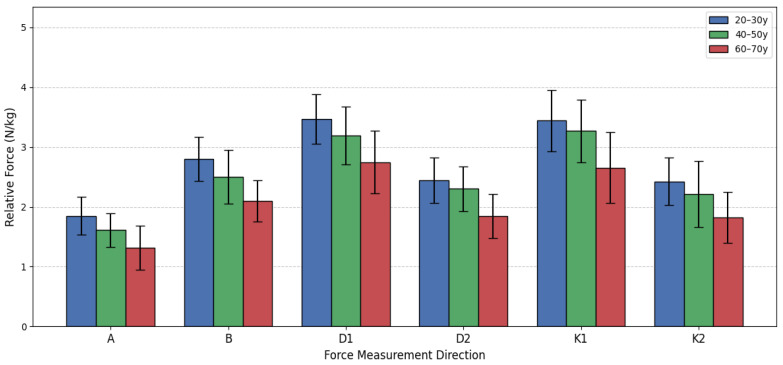
Men’s relative force differences by age group.

A similar pattern was observed in Directions B, D1, D2, K1, and K2 (see Figure 12). In all these movements, the G25 and G45 groups were statistically stronger than the G65 group (with *p* values ranging from *p* < 0.001 to *p* = 0.048). The G65 group consistently showed the lowest mean force, with the G25 group holding the greatest advantage over them, ranging from 26.22% (Direction D1) to 40.97% (Direction A). Moreover, in none of the six directions did the difference between the G25 and G45 groups reach the statistically adjusted level of statistical significance (all *p* values were *p* ≥ 0.052). This finding indicates that, for men, the relative isometric lower extremity force is largely preserved from young adulthood into middle age. The substantial strength reduction, resulting in mean force values 20–40% lower, is almost entirely concentrated in the transition to the 60–70-year bracket. This pattern of decline differs slightly from that observed in women, where the decline began earlier in some movements. The data suggests that while men lose absolute muscle mass and force over time, the most critical functional decline, relative to body mass, begins later than it does in women.

### 4.8. Two-Way ANOVA Analysis

The two-way analysis of variance (ANOVA) for the six relative strength measures were performed, examining the main effects of gender and age group, as well as their interaction (see Table 4). This method was chosen to control the family-wise error rate across multiple group comparisons. Furthermore, the results include the partial eta squared ($\eta_{p}^{2}$) to address the critical point about the effect of the size alongside statistical significance. Presented table consist of 7 columns. First column “Strength Measure” identifies which of the six functional directions of muscle chain strength (MCS) the row of analysis pertains to. Column “Source” identifies the cause of the variance. It includes the factors, their interaction, and the residual error. Total variability attributed to that source is expressed as the sum of the squared (SoS) differences between the group mean and the grand mean (for the factor sources) and their group mean (for the Error/Residual source). A larger SoS means the source explains more total variability. The number of independent pieces of information used to calculate the sum of squares is defined as degree of freedom (df). For a factor, it is the number of levels minus one (e.g., Age Group has 3 levels, so df = 3 − 1 = 2). The test statistic (F) is the ratio of the variance explained by the factor (*MS_Factor_*) to the variance unexplained by the model (*MS_Error_*). A large F-value (typically greater than 1) indicates that the factor explains significantly more variance than random error. The probability that the observed result (or a more extreme result) occurred purely by chance, assuming the null hypothesis is true is defined as (*p*). The value $\eta_{p}^{2}$ represents the proportion of variance in the strength scores that is uniquely attributable to that factor, after controlling for other factors in the model. It measures the practical significance of the finding. Based on the ANOVA results, the analysis revealed a highly significant main effect for both gender and age group on all six lower limb muscle chain strength measures, indicating that strength is substantially affected by sex and decade of life. However, while males are generally stronger than females and both sexes lose strength with age, the rate or trajectory of that strength loss is not significantly different between the sexes in this study population.

## 5. Discussions

The primary objective of this study was to assess leg muscle strength across various age and gender cohorts, providing normative data for static force production in both the frontal and sagittal planes. The results confirm several established physiological principles regarding muscle strength and reveal specific age- and direction-dependent interactions between male and female strength profiles. The most consistent finding across all measurements was that male lower-extremity muscle strength was significantly greater than that of females (*p* < 0.05). Men demonstrated average strength values that were approximately 16.23% to 36.25% higher than women, depending on the specific movement direction tested. This result aligns perfectly with established literature, such as the comprehensive narrative review by James L. Nuzzo [25], which confirms that female muscle strength is consistently lower than male strength across various muscle groups. Nuzzo highlights significant differences, noting that female knee extensor and flexor strength are, on average, 65% and 60% of male strength, respectively. Nuzzo’s comprehensive review often references studies using absolute strength (measured in Newtons or kilograms). Our study exclusively reports relative strength (N/kg). Since men generally have greater body mass and a higher proportion of fat-free mass, normalizing the force to body weight significantly and intentionally reduces the observed gender gap, presenting a more accurate picture of muscle quality and capacity. The physiological basis for this disparity is attributed to anatomical and hormonal factors, including differences in muscle length, muscle cross-sectional area, and the substantial difference in testosterone levels. the lowest difference between genders across the entire analysis was observed during movements in Direction B (leg abduction), where the male advantage ranged from only 16.23% to 18.37%. This finding supports the research by Martin Alberto Belzunce et al. [26], whose work on cyclists suggested that the relative muscle mass of the m. gluteus medius (the muscle primarily responsible for abduction) and other gluteal muscles showed less variance between male and female cyclists. This suggests that the strength disparity may be less pronounced in muscle groups that are highly activated for lateral stabilization or when strength is normalized to muscle size, warranting further investigation into functional versus absolute strength metrics.

Age proved to be a decisive factor, demonstrating a clear and consistent decline in lower-extremity muscle strength with increasing age. The youngest group (20–30 years) consistently exhibited the highest mean strength, while the oldest group (60–70 years) recorded the lowest. The overall analysis showed that the strength of the 20–30-year-old group was approximately 28% to 38% greater than the 60–70-year-old group across most exercise directions. The difference between the young (20–30 years) and middle-aged (40–50 years) groups was relatively smaller, varying between 6% and 13%, and these differences were often not found to be statistically significant. In contrast, the 40–50-year-old group significantly surpassed the 60–70-year-old group by approximately 19% to 25%. These results clearly indicate that muscle strength decreases more slowly in young and middle-aged individuals but accelerates dramatically in older age due to age-related muscle changes and muscle atrophy (sarcopenia). This pattern is consistent with the findings of Gomes M. et al. [27], who noted that muscle atrophy begins around 40 years of age and accelerates nearly twofold after the age of 70, highlighting the critical importance of early strength interventions. While men maintained a strength advantage over women in every age group, the magnitude of the male strength advantage changed with age depending on the direction of the movement. In Direction A (adduction), the strength difference between men and women decreased from the 20–30-year-old group to the 60–70-year-old group. These dynamic changes may be attributable to hormonal shifts associated with aging, such as menopause in women (leading to a sudden drop in estrogen) and the onset of sarcopenia, which may affect muscle groups differently between genders. This observation partially aligns with the research by Ailin Bian [28], who suggests that strength disparities between men and women widen with age due to varying hormonal decline rates (men’s testosterone decreasing gradually, women’s estrogen decreasing sharply during menopause). Bian’s study also points out the increased prevalence of sarcopenia cases registered starting from the 65-year-old age group.

### Study Limitations and Future Research

Our study utilized a convenience sample of 99 non-professional athletes, stratified into three specific age decades (20–30, 40–50, and 60–70 years). This approach means two significant constraints on generalizability, such as, population specificity, size and diversity. The participants were all self-identified non-professional athletes, the resulting normative strength values likely represent a higher-performing, physically active subset of the general population. The established benchmarks should not be extrapolated to sedentary or deconditioned clinical groups, as this would lead to incorrect conclusions regarding rehabilitation goals. The moderate sample size prevents us from establishing robust reference data across finer age stratifications or diverse socioeconomic backgrounds. The most significant constraint is the lack of a specific clinical cohort. While our data successfully establishes normative values for age- and gender-stratified non-professional athletes, we currently lack empirical evidence to confirm the system’s sensitivity and practical utility in a clinical setting.

Since our study collected data from three distinct age groups (20–30, 40–50, and 60–70 years) at a single point in time, our findings can only demonstrate age-related differences between these groups. We can state with confidence that the 60–70 age group, for example, has significantly lower relative strength compared to the 20–30 age group. The differences observed could be partially attributable to other unmeasured factors unique to each group’s cohort, such as lifetime physical activity habits, past injuries, or differences in nutritional intake that accumulated over time. This design prevents us from confidently stating that the difference is purely due to the aging process itself. Therefore, we explicitly temper our conclusions by emphasizing that the observed trends are suggestive, but not definitive, of true longitudinal strength changes. We establish that our results serve as a foundation for, and directly necessitate, future longitudinal studies that track the same participants over decades to accurately quantify the true rate of age-related strength loss in this specific population. The proposed measuring stand represents a significant advancement in musculoskeletal assessment, offering a future for objective rehabilitation metrics. Its key strength lies in providing six precise, standing strength measurements in both the frontal and sagittal planes. This level of functional detail goes far beyond traditional handheld dynamometers, offering insights into stability and dynamic strength essential for walking and balance.

## 6. Conclusions

The findings robustly demonstrate that male leg muscle strength is consistently superior to female strength due to established physiological factors. Furthermore, leg muscle strength decreases significantly with age due to inherent muscular changes. The magnitude of the gender–strength difference changes with age depending on the specific muscle chain activated (frontal vs. sagittal plane), emphasizing the necessity of using age- and gender-stratified data for precise clinical assessment and personalized rehabilitation programs. A statistically significant difference (*p* < 0.05) in leg muscle strength was established between men and women across all six static movement directions, with men consistently demonstrating greater force. The smallest strength disparity between genders was observed in Direction B (external leg muscle chain/abduction), suggesting a lesser gender difference in the functional strength of stabilizing muscle groups. Leg muscle strength consistently decreases with age. The oldest group (60–70 years) invariably exhibited the weakest lower limb muscle strength. The strength difference between the 60–70-year-old group and the 40–50-year-old group was more pronounced than the difference between the 40–50 and 20–30-year-old groups. This confirms that the decline in muscle strength accelerates rapidly in older age, highlighting the need for age-specific rehabilitation targets to counteract sarcopenia. The use of the 3D force sensor fixed in the stable platform significantly reduced testing time and simplified the measurement procedure. This methodological approach proves to be a simple, fast, and reliable method for objectively assessing standing lower limb strength in multiple planes, providing essential normative benchmarks for clinical rehabilitation assessment tailored to specific age and gender cohorts.

## Figures and Tables

**Table 1:** The primary muscles and joints involved in generating force.

Direction	Plane of Action	Primary Joints Involved	Primary Muscle Chain Function	Key Muscle Groups Engaged
A (Lateral Push/Outward)	Frontal	Hip, Knee, Ankle	Hip Abduction and Knee/Ankle Stabilization	Gluteus Medius/Minimus (force generation), Tensor Fasciae Latae (TFL), Core Obliques (for trunk stability).
B (Medial Push/Inward)	Frontal	Hip, Knee, Ankle	Hip Adduction and Knee/Ankle Stabilization	Adductor Magnus, Longus, and Brevis, Pectineus (force generation), Core Stabilizers (to prevent lateral sway).
D1 (Forward Push)	Sagittal/Diagonal	Hip, Knee, Ankle	Hip & Knee Extension (similar to a squat or skate push-off)	Gluteus Maximus, Quadriceps (Vasti muscles, Rectus Femoris), Calf Muscles (Gastrocnemius/Soleus), Erector Spinae (for trunk extension).
D2 (Backward Pull)	Sagittal/Diagonal	Hip, Knee, Ankle	Hip & Knee Flexion (similar to a leg-curl or deadlift stance stabilization)	Hamstrings (Biceps Femoris, Semitendinosus, Semimembranosus), Hip Flexors (Iliopsoas, Rectus Femoris), Core Abdominals (for trunk flexion stability).
K1 (Diagonal Push-In)	Diagonal/Mixed	Hip, Knee, Ankle	Hip Internal Rotation and Adduction (pushing inward/forward)	Combination of Adductors, Quadriceps, Gluteus Medius/Minimus (Anterior fibers), and Tibialis Posterior (for foot/ankle stability).
K2 (Diagonal Pull-Out)	Diagonal/Mixed	Hip, Knee, Ankle	Hip External Rotation and Abduction (pulling outward/backward)	Combination of Gluteus Maximus/Medius (Posterior fibers), External Rotators (Piriformis, Gemelli, Obturators), and Hamstrings (stabilizing the knee).

**Table 2:** Description of movements of lower limbs.

Direction	Movement Description	Muscle Chains Assessed
Direction A	Inward Pull (Adduction): The participant stands on the measuring stand and pulls the sides of the stand inward toward the center.	Inner leg muscle chains (primarily Hip Adductors and related musculature).
Direction B	Outward Push (Abduction): The participant stands on the measuring stand and pushes the sides of the stand outward away from the center.	Outer leg muscle chains (primarily Hip Abductors and related musculature).

**Table 3:** Anthropometric characteristics by group.

Group	Height Range (cm)	Height Mean (SD)	Mass Range (kg)	Mass Mean (SD)	BMI Range	BMI Mean (SD)
G25 Men	175–205	186.47 (8.50)	61–117	86.11 (15.52)	19.66–37.24	22.21 (3.32)
G25 Women	158–183	168.22 (7.29)	44–91	63.00 (11.12)	16.36–30.06	22.21 (3.32)
G45 Men	176–202	186.35 (8.05)	75–123	91.53 (15.91)	21.13–37.65	26.41 (4.68)
G45 Women	160–175	168.67 (15.95)	53–95	71.07 (10.99)	18.78–32.11	24.93 (3.35)
G65 Men	169–186	177.27 (5.58)	62–106	81.27 (12.83)	19.75–33.83	25.84 (3.79)
G65 Women	162–179	168.80 (5.04)	59–98	71.00 (11.05)	19.71–31.64	25.07 (3.59)

**Table 4:** The ANOVA tables for each of the six Lower Limb Muscle Chain Strength (MCS) directions.

Strength Measure	Source	SoS	df	F	*p*	$\eta_{p}^{2}$
Lateral Pull (A)	Gender	3.64	1	39.970	0.0000	0.301
	Age Group	2.95	2	16.175	0.0000	0.258
	Interaction (Gender × Age)	0.23	2	1.265	0.2870	0.026
	Error (Residual)	8.47	93	NaN	NaN	NaN
Lateral Push (B)	Gender	3.14	1	20.806	0.0000	0.183
	Age Group	6.85	2	22.671	0.0000	0.328
	Interaction (Gender × Age)	0.06	2	0.205	0.8149	0.004
	Error (Residual)	14.05	93	NaN	NaN	NaN
Forward Push (D1)	Gender	7.44	1	39.636	0.0000	0.299
	Age Group	8.11	2	21.588	0.0000	0.317
	Interaction (Gender × Age)	0.01	2	0.026	0.9747	0.001
	Error (Residual)	17.46	93	NaN	NaN	NaN
Backward Pull (D2)	Gender	6.28	1	54.439	0.0000	0.369
	Age Group	5.94	2	25.713	0.0000	0.356
	Interaction (Gender × Age)	0.14	2	0.602	0.5496	0.013
	Error (Residual)	10.73	93	NaN	NaN	NaN
Diagonal Push-In (K1)	Gender	8.42	1	35.375	0.0000	0.276
	Age Group	10.16	2	21.355	0.0000	0.315
	Interaction (Gender × Age)	0.04	2	0.087	0.9172	0.002
	Error (Residual)	22.12	93	NaN	NaN	NaN
Diagonal Pull-Out (K2)	Gender	5.57	1	35.478	0.0000	0.276
	Age Group	6.18	2	19.685	0.0000	0.297
	Interaction (Gender × Age)	0.02	2	0.058	0.9432	0.001
	Error (Residual)	14.59	93	NaN	NaN	NaN

## Data Availability

The data will be made available by the corresponding author on request.
